# A ruthenium(II)-curcumin compound modulates NRF2 expression balancing the cancer cell death/survival outcome according to p53 status

**DOI:** 10.1186/s13046-020-01628-5

**Published:** 2020-06-30

**Authors:** Alessia Garufi, Silvia Baldari, Riccardo Pettinari, Maria Saveria Gilardini Montani, Valerio D’Orazi, Giuseppa Pistritto, Alessandra Crispini, Eugenia Giorno, Gabriele Toietta, Fabio Marchetti, Mara Cirone, Gabriella D’Orazi

**Affiliations:** 1 Department of Research and Advanced Technologies, IRCCS Regina Elena National Cancer Institute, Rome, Italy; 2 University “G. D’Annunzio”, School of Medicine, Chieti, Italy; 3 Department of Medical, Surgical Sciences, and Biotechnologies, Sapienza University, Latina, Italy; 4 School of Pharmacy, Chemistry Section, University of Camerino, Camerino Macerata, Italy; 5 Department of Experimental Medicine, Sapienza University, laboratory affiliated to Pasteur Institute Italy Foundation Cenci Bolognetti, Rome, Italy; 6 Department of Surgical Sciences, Sapienza University, Rome, Italy; 7 Italian medicines agency-Aifa, centralized procedure office, Rome, Italy; 8 Department of Chemistry and Chemical Technologies, laboratory MAT-IN LAB, Calabria University, Rende, Italy; 9 School of Science and Technology, Chemistry Section, University of Camerino, Camerino Macerata, Italy

**Keywords:** p53, NRF2, Curcumin, (arene)ruthenium(II) compound, Brusatol, Cancer therapy, Oxidative stress, Chemoresistance, Autophagy

## Abstract

**Abstract:**

**Background:**

Tumor progression and tumor response to anticancer therapies may be affected by activation of oncogenic pathways such as the antioxidant one induced by NRF2 (nuclear factor erythroid 2-related factor 2) transcription factor and the pathways modified by deregulation of oncosuppressor p53. Often, oncogenic pathways may crosstalk between them increasing tumor progression and resistance to anticancer therapies. Therefore, understanding that interplay is critical to improve cancer cell response to therapies. In this study we aimed at evaluating NRF2 and p53 in several cancer cell lines carrying different endogenous p53 status, using a novel curcumin compound since curcumin has been shown to target both NRF2 and p53 and have anti-tumor activity.

**Methods:**

We performed biochemical and molecular studies by using pharmacologic of genetic inhibition of NRF2 to evaluate the effect of curcumin compound in cancer cell lines of different tumor types bearing wild-type (wt) p53, mutant (mut) p53 or p53 null status.

**Results:**

We found that the curcumin compound induced a certain degree of cell death in all tested cancer cell lines, independently of the p53 status. At molecular level, the curcumin compound induced NRF2 activation, mutp53 degradation and/or wtp53 activation. Pharmacologic or genetic NRF2 inhibition further increased the curcumin-induced cell death in both mutp53- and wtp53-carrying cancer cell lines while it did not increase cell death in p53 null cells, suggesting a cytoprotective role for NRF2 and a critical role for functional p53 to achieve an efficient cancer cell response to therapy.

**Conclusions:**

These findings underline the prosurvival role of curcumin-induced NRF2 expression in cancer cells even when cells underwent mutp53 downregulation and/or wtp53 activation. Thus, NRF2 inhibition increased cell demise particularly in cancer cells carrying p53 either wild-type or mutant suggesting that p53 is crucial for efficient cancer cell death. These results may represent a paradigm for better understanding the cancer cell response to therapies in order to design more efficient combined anticancer therapies targeting both NRF2 and p53.

## Background

The oncosuppressor p53 plays a key role in cell growth and apoptosis in response to various stress signals [1]. Given its central role in maintaining genomic stability and preventing oncogenesis, p53 is the most inactivated oncosuppressor in human tumors by gene mutations or by protein deregulation [2]. Mutant (mut) p53 proteins may acquire a misfolded hyperstable conformation [3] that may be achieved by binding heat shock proteins (HSP) such as HSP90, a cellular chaperone that is crucial for the stability of many client proteins including mutp53 [4, 5]. Besides loss of function and dominant-negative effect on the wild-type (wt) p53 activity, the hotspot p53 mutants may also acquire new oncogenic functions, contributing to cancer progression, invasion and resistance to therapies [6]. Thus, targeting mutp53 is a challenging strategy to halt cancer growth [7]. In this regard, several different approaches have been taken in the last years developing small molecule or using phytochemicals from nature to induce mutp53 degradation or conformational changes, providing new insight on mutp53 reactivation [8, 9], as also demonstrated by our studies [10–13]. Autophagy has been shown to be involved in mutp53 degradation [14–23], suggesting the use of autophagy stimulators to counteract mutp53 oncogenic activity. Thus, mutp53 has been shown to counteract autophagy mechanism to likely halt its own degradation [24]. Finally, mutp53 degradation by autophagy has been shown to increase the cytotoxic effects of chemotherapeutic drugs [17]. Mutp53 oncogenic activities ma also depend by modifications of the tumor microenvironment altering the secretion of inflammatory cytokines that affect the crosstalk between cancer and stromal cells [25, 26] or by interaction with other transcription factors such as NRF2 (nuclear factor erythroid 2-related factor 2, encoded by NFE2L2 gene) or HIF-1 (hypoxia-inducible factor 1) to support tumor progression and resistance to therapies [27]. Therefore, understanding the interplay between these oncogenic pathways may have an impact on the development of more efficient targeted anticancer therapies.

NRF2 is the main regulator of cellular antioxidant response [28] and is activated in response to oxidative and/or electrophilic stress, the so-called canonical conditions. Following activation, NRF2 detaches from its negative regulator KEAP1 (Kelch-like ECH-associated protein 1), stabilizes and moves to the nucleus where it binds to sequence-specific responsive elements of anti-oxidant target genes promoters. Among these genes there are catalase, superoxide dismutase (SOD), HO-1 (heme-oxygenase 1), NAD(P)H quinone oxidoreductase 1 (NQO1), and glutathione (GSH), that help to restore the cellular redox homeostasis [29]. Constitutive activation of NRF2 is found in several different tumors also by gain-of-function mutations of the NFE2L2 gene or by inactivating mutations of the KEAP1 gene. These mutations are considered drivers of cancer progression, metastasis, and resistance to therapies [30]. NRF2 noncanonical activation may depend by p62/SQSTM1-mediated KEAP1 degradation [31], or by p21Cip1/WAF1 (target of p53) that binds to KEAP1 to interrupt the KEAP1/NRF2 complex [32]. NRF2 may have both tumor suppressive and tumor-promoting actions and is therefore considered a “double face” molecule [33]. Thus, while NRF2 transient activation is certainly considered cytoprotective, its continual activation may support tumor progression and tumor resistance to therapies. Therefore, NRF2 overexpression in cancer cells may be considered a marker of chemoresistance [34].

Curcumin is considered a chemopreventive molecule with anti-oxidant, anti-apoptotic and anti-inflammatory properties and with an excellent safety profile although it presents low solubility and bioavalibility [35]. Curcumin has been shown to activate the NRF2 pathway triggering cellular protection against oxidative injury that, in advanced stage cancers, may induce chemoresistance [36]. On the other hand, curcumin may induce mutp53 degradation through autophagy or convert mutp53 protein to transcriptionally active wtp53, reversing the therapeutic resistance and improving cancer cell death, as also demonstrated by our studies [16–18, 37, 38].

In this study we aimed at evaluating the effect of a novel curcumin compound in several cancer cell lines carrying different endogenous p53 status. We used a water-soluble ruthenium(II)-curcumin (RuCUR) compound that presents high solubility and cytotoxic effect ]compared to other similar curcumin compounds [39–46]. Moreover, the compound show selectivity toward cancer cells over nontumorous cells, suggesting an in vivo use [42]. We found that RuCUR induced cancer cell death in all tested cell lines and independently of the p53 status. At molecular level we found NRF2 activation, mutp53 degradation and/or wtp53 activation. Pharmacologic or genetic NRF2 inhibition further increased the RuCUR-induced cell death in both mutp53- and wtp53-carrying cancer cell lines while did not increase cell death in p53 null cells. In the present study, we found that activation of the NRF2 antioxidant pathway contributed to resistance to RuCUR treatment, despite mutp53 degradation and activation of wtp53. Furthermore, inhibition of NRF2 could overcome this resistance in particular in cancer cells carrying p53 either wild-type or mutant, indicating the crucial role for a functional p53 pathway to efficiently induce cell death in response to treatments.

## Methods

### Ruthenium(II)-curcumin compound (RuCUR) synthesis

**Fig. 1:**
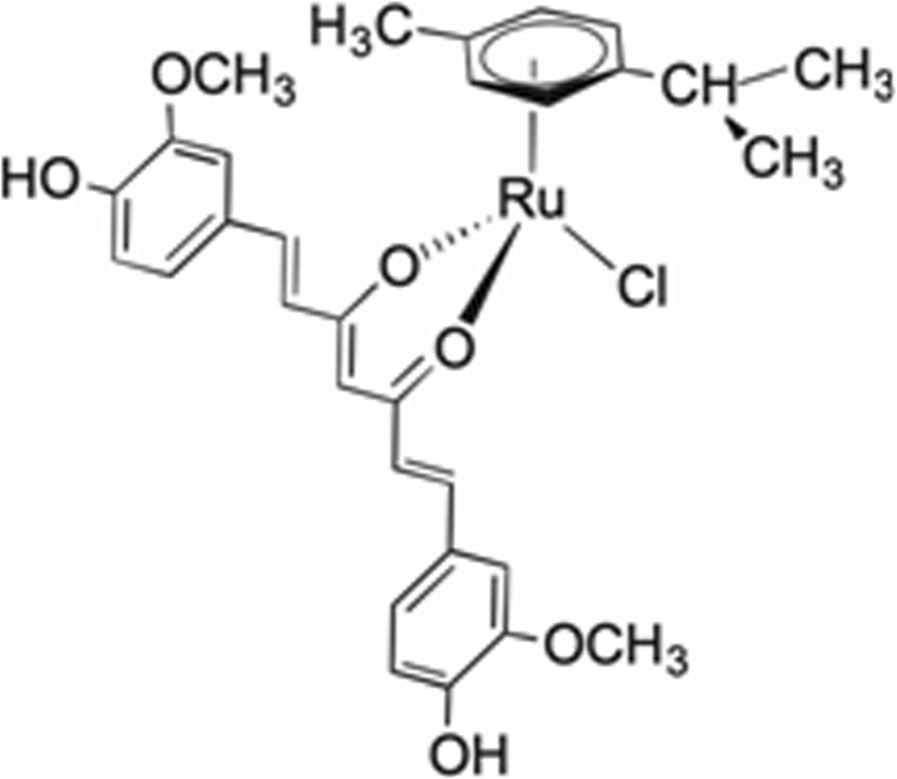
Molecular structure of the compound RuCUR

The water-soluble ruthenium(II)-curcumin compound (RuCUR) was synthesized as described previously [42, 43]. In brief, for the synthesis of RuCUR, equimolar amounts of curcumin and [Ru(6-p-cymene)Cl2]2 were stirred in methanol in the presence of KOH, the mixture was filtered to remove potassium chloride, then the solution was concentrated and stored at 4 °C, from which red crystals of RuCUR slowly formed (Fig. 1). The purity of the prepared product was confirmed through elemental analysis (on a Fisons Instruments 1108 CHNSO elemental analyzer), melting point (on a STMP3 Stuart scientific instrument and on a capillary apparatus), IR spectral analysis (on a PerkinElmer Frontier FT-IR instrument) and 1H and 13C NMR spectroscopy (on a 500 Bruker Ascend operating at 500 MHz for 1H and 125 MHz for 13C relative to TMS). The RuCUR compound was dissolved in water and used at different concentration.

### Cell culture and reagents

The cell lines used in this study were: human SKBR3 (breast cancer, carrying R175H p53 mutation), T98MG (glioblastoma, carrying M237I p53 mutation), MCF7 breast cancer, U87 glioblastoma, HCT116 and RKO colon cancer cell lines (all carrying wild-type p53), and HCT116 p53 null. The cells were cultured in either DMEM (Dulbecco modified Eagle’s medium) (Life Technologies-Invitrogen), or RPM1–1640 (Life Technologies-Invitrogen), with 10% heat-inactivated foetal bovine serum (FBS) (Corning, NY, USA, #35–079) and L-glutamine/streptomycin (100 μg/mL) (Corning, NY, USA, #30–002), in 5% CO2 at 37 °C. They were all mycoplasma negative. The NRF2 inhibitor Brusatol [47] (Sigma-Aldrich) was used at 100 nM, as previously reported [48].

### Viability assay

For viability assay cells were plated at subconfluence in 35 mm multiwall Petri dishes and the day after treated as reported. Both floating and adherent cells were collected and cell viability was determined by Trypan blue (Sigma-Aldrich, #72571) staining and direct counting with a Neubauer haemocytometer, as previously reported [13].

### 3D spheroids proliferation assay

The 3D spheroids, were obtained into ultra-low attachment cell culture multiwell plates (96 well, Corning), as previously reported [49, 50]. When spheroids reached approximate size of 300–500 um (about 4 days after platinga), they were treated with RuCUR (10, 50, 100 μM). Spheroids images were acquired with a Nikon Eclipse TS100 microscope equipped with a Nikon ELWD camera (Nikon Instrument Europe BV, Amsterdam, The Netherlands) every 24 h. The formula to evaluate the spheroids volume was: V = a x (b2)/2, where a and b are, respectively, length and width.

### Measurement of intracellular Reactive Oxygen Species (ROS)

Reactive oxygen species (ROS) production was assessed by using the 2′,7′-dichlorofluorescein diacetate (DCFDA; Sigma–Aldrich) staining, as previously reported [51]. Cell pellets were collected and analyzed in the FL-1 channel of a FACScalibur flow cytometer (BD Transduction Laboratories), using CELLQuest Pro software (version 6.0, BD Biosciences). DCF fluorescence values were expressed as mean fluorescence intensity (MFI).

### Western blot analysis

Cell pellets were lysed in lysis buffer (50 mM Tris–HCl, pH 7.5, 150 mM NaCl, 5 mM EDTA, 150 mM KCl, 1 mM dithiothreitol, 1% Nonidet P-40) (all from Sigma-Aldrich) and a mix of protease inhibitors (Complete™, Mini Protease Inhibitor Cocktail, Merk, Life Science S.r.l., Milan, Italy, #11836153001) and protein concentration determined with the BCA Protein Assay (BioRad, Hercules, CA, USA) to load equal amount of total cell extracts on 9–18% SDS-polyacrilamide gels (PAGE) (Bio-Rad, #456–1095) electrotrophoreses. After blotting to polyvinylidene difluoride membranes (PVDF, Millipore Corporation, Billerica, MA, USA, #IPVH 00010). Unspecific sites were blocked with 5% non-fat powdered milk or with 3% bovine serum albumin (BSA, both from Sigma-Aldrich) in 0.05% Tween-20 (v/v in TBS). Membranes were then incubated with primary antibodies. The antibodies we used in this study were: mouse monoclonal anti-p53 (DO-1) (1:1000) (sc-126), rabbit polyclonal anti-p21 (C-19) (1:500) (sc-397), mouse monoclonal anti-HO-1 (A-3) (1:1000) (sc-136,960), mouse monoclonal anti-p62/SQSTM1 (D-3) (1:1000) (sc-28,359), all from Santa Cruz Biotechnology; rabbit polyclonal anti-LC3B (1:2000) (Sigma, #L7543); mouse monoclonal anti-NQO1 (A180) (ThermoFisher, #39–3700), mouse monoclonal anti-HSP90 (1:1000) (BD Bioscience, #610418), rabbit polyclonal anti-NRF2 (1:1000) (Abcam, #ab62352), mouse monoclonal anti-poly(ADP-ribose) polymerase (1:1000) (PARP, cleavage site-214-215, Sigma, #AB3565), mouse monoclonal anti-phospho-Histone H2AX (1:1000) (Ser139, clone JBW301) (Sigma, #05–636), and rabbit polyclonal anti phospho-4E-BP1 (236B4) (Thr37/46, Cell Signaling Technology, #2855). Mouse monoclonal anti-actin antibody (Ab-1) (JLA20) (1:10.000) (Calbiochem, #CP01) was used as loading control. Anti-imunoglobulin-G-horseradish peroxidise (HRP) secondary antibodies that we used were: anti-mouse IgG-HRP (BioRad, #172–1011) and anti-rabbit IgG-HRP (BioRad, #172–1019). Enzymatic signals were visualized by chemiluminescence (ECL Detection system, Amersham GE Healthcare, Milan, Italy, #RPN2106), according to the manufacturer’s protocol.

### RNA extraction and semi-quantitative reverse transcription (RT)-PCR analysis

Total RNA extraction was performed by using TRIzol Reagent (Life Technology-Invitrogen), cDNA was synthesized by using MuLV reverse transcriptase kit (Applied Biosystems, Foster City, CA, USA) and semi-quantitative Reverse-Transcribed (RT)-PCR was carried out by using Hot-Master Taq polymerase (Eppendorf, Milan, Italy), as previously reported [13]. Densitometric analysis allowed to quantify mRNA levels compared to the control 28S gene expression. Primer sequences are as follow: NRF2 For: TCCATTCCTGAGTTACAGTGTCT; Rev.: TGGCTTCTGGACTTGGAACC. HO-1 For: AAGATTGCCCAGAAAGCCCTGGAC; Rev.: AACTGTCGCCACCAGAAAGCTGAG. DRAM For: TCAAATATCACCATTGATTTCTGT; Rev.: GCCACATACGGATGGTCATCTCTG. PUMA For: TGTGAATCCTGTGCTCTGCC; Rev.: TTCCGGTATCTACAGCAGCG. P21 For: CCCCTTCGGCCCGGTGGAC; Rev.: CCGTTTTCGACCCTGAGAG. 28S For: GTTCACCCACTAATAGGGAACGTGA; Rev.: GATTCTGACTTAGAGGCGTTCAGT.

### siRNA interference

For RNA interference, cells were transfected with the Nrf2 siRNA (sc-3703, Samta Cruz Biotechnology) and control siRNA (sc-37,007, Santa Cruz Biotechnology) using LipofectaminePLus reagent (Invitrogen, #11514–015), following the manufacturer’s instructions, as previously reported [12].

### Densitometric analyis

Quantification of the protein bands was assessed by densitometric analysis using the ImageJ software (http://imagej.nih.gov) and relative band intensity normalized to β-actin signals.

### Statistical analysis

Data are presented as mean ± standard deviation (S.D.) of at least three independent experiments. Two-tailed Student’s t-test and one-way ANOVA analysis were used for statistical significance of, respectively, two o more sample comparisons. Difference was considered statistical significant when *p*-value was ≤0.05.

## Results

### RuCUR compound induces mutp53 downregulation and cell death

**Fig. 2:**
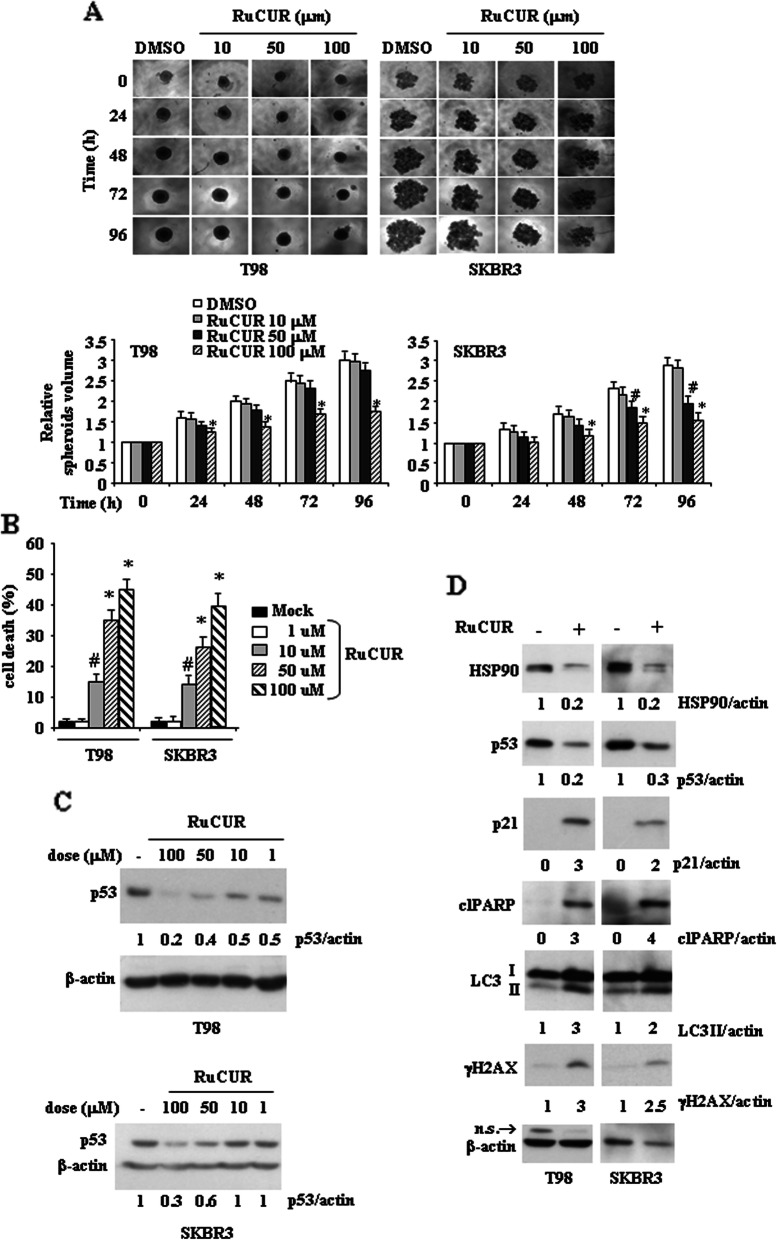
RuCUR (ruthenium-curcumin) compound reduced mutant (mut) p53 levels that correlated with reduced proliferation and increased cell death. (**a**) Mutp53-carrying T98 and SKBR3 cells were seeded on ultra-low attachment multiwell plates allowing for tumor spheroid formation. Four days after seeding, spheroids were formed (approximate size 300–500 μm) and then treated with different doses of RuCUR (10, 50, 100 μM) for the indicated times. Tumor spheroids volume was quantified according to the formula: V = a x (b2)/2, where a and b are, respectively, length and width. Representative images of spheroids derived from both cell lines are shown in the upper panels. Spheroids volumes are reported on the bottom panels. Histograms represent the fold increase quantified with respect to controls set to 1.0, ± SD. * (*p* ≤ 0.01), # (*p* ≤ 0.05) (single treatments compared to untreated spheroids). (**b**) Cell viability was measured by trypan blue exclusion assay in T98 and SKBR3 cells treated for 24 h with different doses of RuCUR (1, 10, 50, 100 μM) and expressed as cell death percentage ± S.D. * (*p* ≤ 0.01), # (*p* ≤ 0.05) (single treatments compared to untreated cells). (**c**) Western blot analysis of p53 protein was performed in T98 and SKBR3 cells untreated or treated with RuCUR (1, 10, 50, 100 μM) for 24 h. The ratio of p53 levels vs β-actin, following densitometric analysis using ImageJ software, is shown. (**d**) Western blot analysis of the indicated protein levels was performed in T98 and SKBR3 cells untreated or treated with RuCUR (100 μM) for 24 h. Actin was used as protein loading control. The ratio of the protein levels vs β-actin, following densitometric analysis using ImageJ software, is reported. n.s = not specific

We first evaluated the effect of RuCUR compound on cancer cell lines carrying mutp53 by performing a three-dimensional (3D) culture spheroids proliferation assay [49, 50]. Cells cultured in ultralow attachment plates were treated with different doses of RuCUR compound and spheroids proliferation was recorded acquiring images every 24 h. Figure 2a shows that increasing doses of RuCUR significantly inhibited spheroids proliferation, indicative of the capacity of the compound to reach cells even in 3D culture conditions, and, accordingly, induced cell death, as assessed by viability assay (Fig. 2b). At biochemical level, RuCUR reduced mutp53 protein levels at almost all doses although the highest dose was more efficient (Fig. 2c). Therefore, we used the highest dose for the next experiments. We found that RuCUR-induced mutp53 downregulation correlated with strong reduction of HSP90 levels and with induction of autophagic marker LC3II (Fig. 2d); moreover, RuCUR induced p21 expression, indicative of wtp53 reactivation and increased the expression of pro-apoptotic and DNA-damage response proteins (respectively, cleaved PARP and γH2AX) (Fig. 2d). Altogether, these findings suggest that the RuCURC-triggered mutp53 downregulation, likely through autophagy, correlated with unbalance of the cell death/survival pathways toward cell death.

### RuCUR compound induces cell death in wild-type (wt) p53-carrying cancer cells

**Fig. 3:**
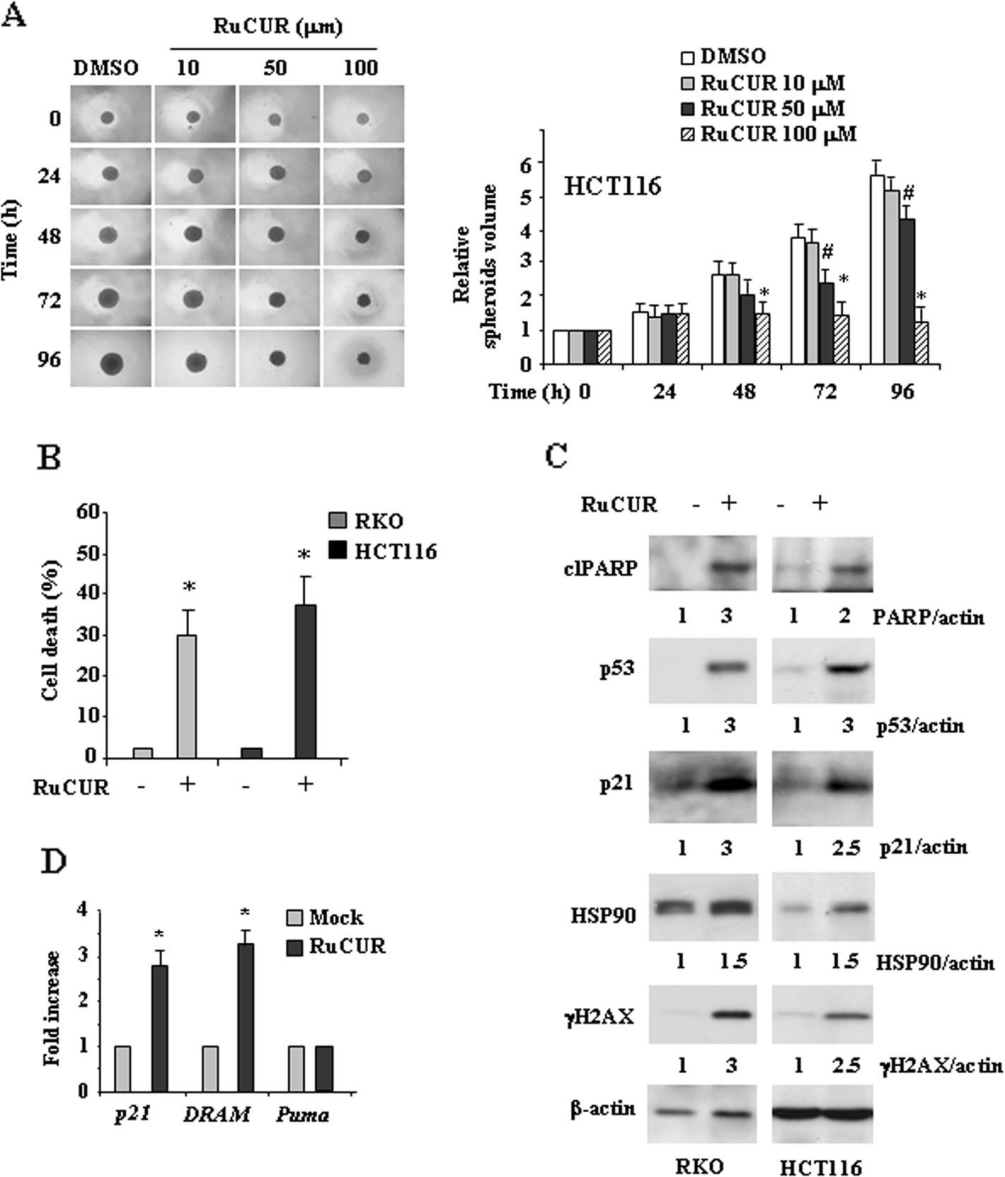
RuCUR compound reduces proliferation and induces cell death in wild-type (wt) p53-carrying cancer cells. (**a**) Colon cancer HCT116 cell, were seeded on ultra-low attachment multiwell plates allowing for tumor spheroid formation. Four days after seeding, spheroids were formed (approximate size 300–500 μm) and then treated with different doses of RuCUR (10, 50, 100 μM) for the indicated times. Tumor spheroids volume was quantified according to the formula: V = a x (b2)/2, where **a** and **b** are, respectively, length and width. Representative images of spheroids are shown in left panel. Spheroid volume are reported on the right panel. Histograms represent the fold increase quantified with respect to controls set to 1.0, ± SD. * (*p* ≤ 0.01), # (*p* ≤ 0.05) (single treatments compared to untreated spheroids). (**b**) Cell viability was measured by trypan blue exclusion assay in RKO and HCT116 cells treated with RuCUR (100 μM) for 24 h and expressed as cell death percentage ± S.D. * (*p* ≤ 0.01) (single treatments compared to untreated cells). (**c**) Western blot analysis of the indicated protein was performed in RKO and HCT116 cells untreated or treated with RuCUR (100 μM) for 24 h. Actin was used as protein loading control. The ratio of the protein levels vs β-actin, following densitometric analysis using ImageJ software, is reported. (**d**) Total mRNA was extracted from RKO and HCT116 cells treated with RuCUR (100 μM) for 24 h. The indicated gene expression was assayed by the polymerase chain reaction (PCR) of reverse-transcribed cDNA. Densitometric analysis was performed using ImageJ software to calculate the gene/28S ratio. Histograms represent the fold increase quantified with respect to controls set to 1.0, ± SD. * (*p* ≤ 0.01)

Next we evaluated the effect of RuCUR compound on cancer cell lines carrying wtp53. We found that increasing doses of RuCUR compound significantly inhibited spheroids proliferation (Fig. 3a) and, accordingly, induced cell death as assessed by viability assay (Fig. 3b). At biochemical level RuCUR induced cleavage of PARP and p53 stabilization with up-regulation of the levels of its targets p21 and DRAM, although it did not induce the pro-apoptotic Puma target gene (Fig. 3c, d), suggesting a selective and not completely apoptotic p53 activation, as previously reported [52]. RuCUR induced histone H2AX phosphorylation, producing γH2AX, indicative of DNA damage response, responsible of p53 activation (Fig. 3c). Finally, differently from what observed for mutp53 cells in Fig. 2d, RuCUR slightly increased HSP90 levels in wtp53-carrying cells (Fig. 3c), in agreement with the finding that HSP90 can promote p53 transcriptional activity [53] and that curcumin may indirectly target HSP90 according to cell type [54]. Altogether, these results indicate that RuCUR-induced cell death in wtp53 cells correlated with wtp53 activation.

### RuCUR compound induces NRF2 pathway in mutp53-carrying cancer cells

**Fig. 4:**
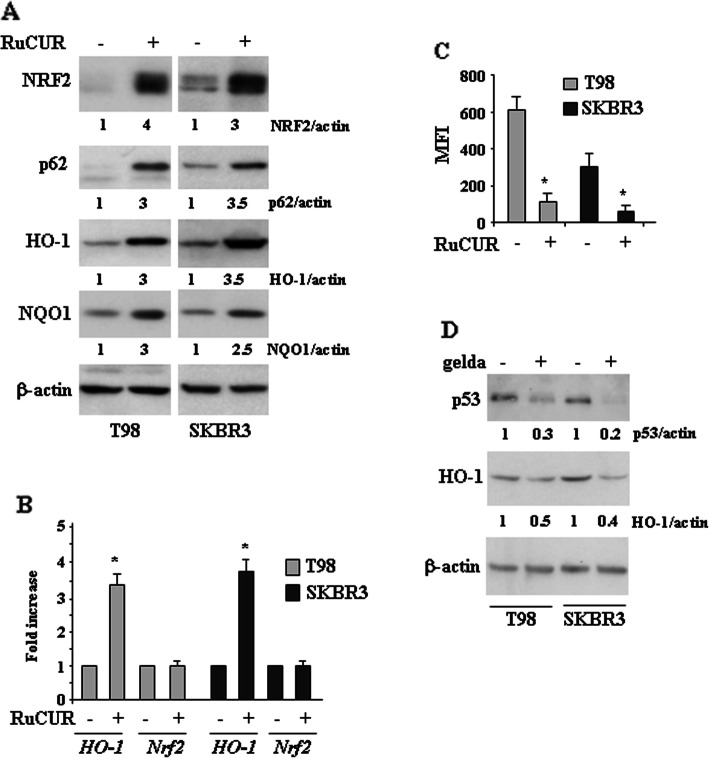
RuCUR compound induces NRF2 pathway in mutp53-carrying cancer cells. (**a**) Western blot analysis of the indicated protein was performed in T98 and SKBR3 cells untreated or treated with RuCUR (100 μM) for 24 h. Actin was used as protein loading control. Densitometry was performed using ImageJ software. Relative band intensities value were normalized to β-actin (loading control) and finally quantified with respect to untreated control arbitrarily set to 1.0. (**b**) Total mRNA was extracted from T98 and SKBR3 cells untreated or treated with RuCUR (100 μM) for 24 h. The indicated gene expression was assayed by semi-quantitative polymerase chain reaction (PCR) of reverse-transcribed cDNA. Densitometric analysis was performed using ImageJ software to calculate the gene/28S ratio. Histograms represent the fold increase quantified with respect to controls set to 1.0, ± SD. * (*p* ≤ 0.01). (**c**) Oxidant species production in T98 and SKBR3 cells after RuCUR (100 μM) treatment for 16 h evaluated by 2′,7′-dichlorofluorescein diacetate (DC-FDA) staining and assessed by Fluorescence-Activated Cell Sorting (FACS) analysis. Histograms of the mean fluorescence intensity (MFI) are the mean ± SD of three independent experiments. * (*p* ≤ 0.01). (**d**) Western blot analysis of the indicated protein levels was performed in T98 and SKBR3 cells untreated or treated with geldanamycin (gelda) (100 nM) for 24 h. The ratio of p53 and HO-1 levels vs β-actin, following densitometric analysis using ImageJ software, is shown. Representative images are shown

Since curcumin has been shown to activate NRF2 [36] we evaluated the effect of RuCUR on NRF pathway in mutp53 cancer cells. We found that RuCUR strongly stabilized the levels of NRF2 protein and induced the expression of its antioxidant targets, including HO-1 and NQO1 by western blot (Fig. 4a) and RT-PCR (Fig. 4b) analyses. Interestingly, increased NRF2 levels correlated with increased p62 levels (Fig. 4a), in according with the notion that p62 competitively binds to Keap1 to activate NRF2 in a noncanonical manner [55]. In support of this, NRF2 gene did not undergo modification following RuCUR treatment (Fig. 4b). Finally, NRF2 stabilization correlated with a strong decrease of intracellular ROS in both cell types (Fig. 4c), in line with the antioxidant effect of NRF2. Since NRF2 and mutp53 may regulate each other [56] we attempted to evaluate the NRF2/mutp53 interplay by using the HSP90 inhibitor geldanamycin [57]. We found that geldanamycin while strongly reduced mutp53 protein levels it did not induce NRF2 pathway but rather decreased HO-1 levels (Fig. 4d), suggesting that targeting mutp53 may inhibit also NRF2 pathway. This did not happen with RuCUR that instead exerted a strong dual effect by both inducing NRF2 and reducing mutp53, suggesting how different molecular approaches may differently modulate the two oncogenic pathways.

**Fig. 5:**
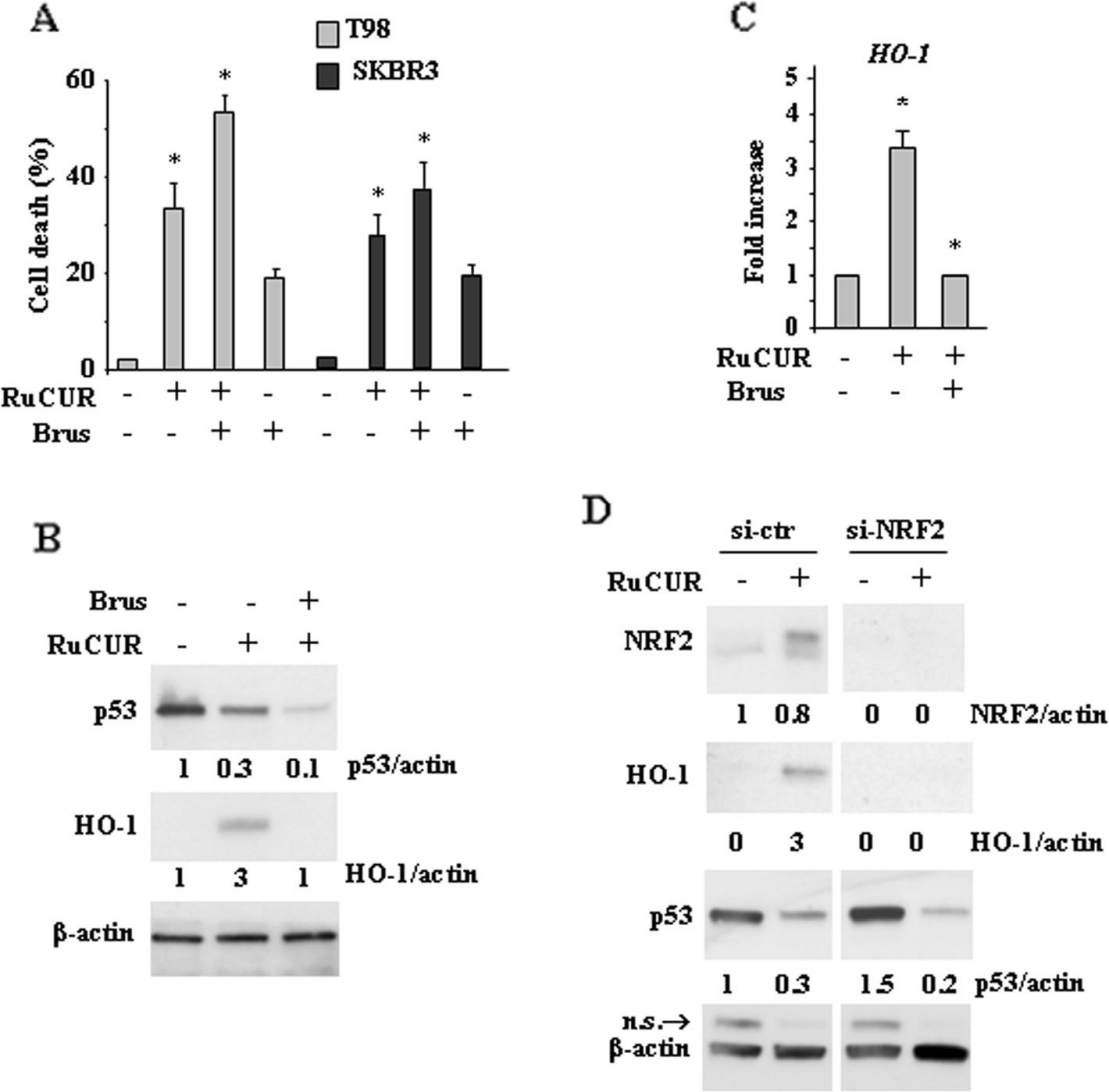
Targeting NRF2 pathway increases cell death of mutp53-carrying cancer cells. (**a**) Cell viability was measured by trypan blue exclusion assay in T98 and SKBR3 cells pre-treated with brusatol (100 nM for 4 h) prior to adding RuCUR (100 μM) for 24 h and expressed as cell death percentage ± S.D. * (*p* ≤ 0.01) (single treatments compared to untreated cells). (**b**) Western blot analysis of the indicated protein in T98 cells pre-treated with brusatol (100 nM for 4 h) prior to adding RuCUR (100 μM) for 24 h. Actin was used as protein loading control. The ratio of protein levels vs β-actin, following densitometric analysis using ImageJ software, is reported. (**c**) Total mRNA was extracted from T98 cells pre-treated with brusatol (100 nM for 4 h) prior to adding RuCUR (100 μM) for 24 h. HO-1 gene expression was assayed by the polymerase chain reaction (PCR) of reverse-transcribed cDNA. Densitometric analysis was performed using ImageJ software to calculate the gene/28S ratio. Histograms represent the fold increase quantified with respect to controls set to 1.0, ± SD. * (*p* ≤ 0.01). (**d**) Western blot analysis of the indicated protein in NRF2-silenced and in siRNA-control (ctr) T98G cells, treated with RuCUR (100 μM) for 24 h. Actin was used as protein loading control. The ratio of the protein levels vs β-actin, following densitometric analysis using ImageJ software, is reported

Since NRF2 is an important pro-survival molecule we attempted to inhibit it. We found that NRF2 pharmacologic inhibition with brusatol [47, 48] significantly increased RuCUR-induced cell death of mutp53 cancer cells, as assessed by viability assay (Fig. 5a). Pharmacologic inhibition of NRF2 activity indeed impaired the RuCUR-induced HO-1 protein levels (Fig. 5b) and gene expression (Fig. 5c). Interestingly, the RuCUR-reduced mutp53 protein levels were further reduced following NRF2 inhibition (Fig. 5b), strengthening the notion that NRF2 and mutp53 may regulate each other [56, 58]. Similar results were obtained with genetic NRF2 silencing using specific siRNA (Fig. 5d). These findings indicate that inhibiting the NRF2 survival pathway further reduces mutp53 levels and increases cancer cell death.

### RuCUR compound induces NRF2 pathway in wtp53-carrying cancer cells

**Fig. 6:**
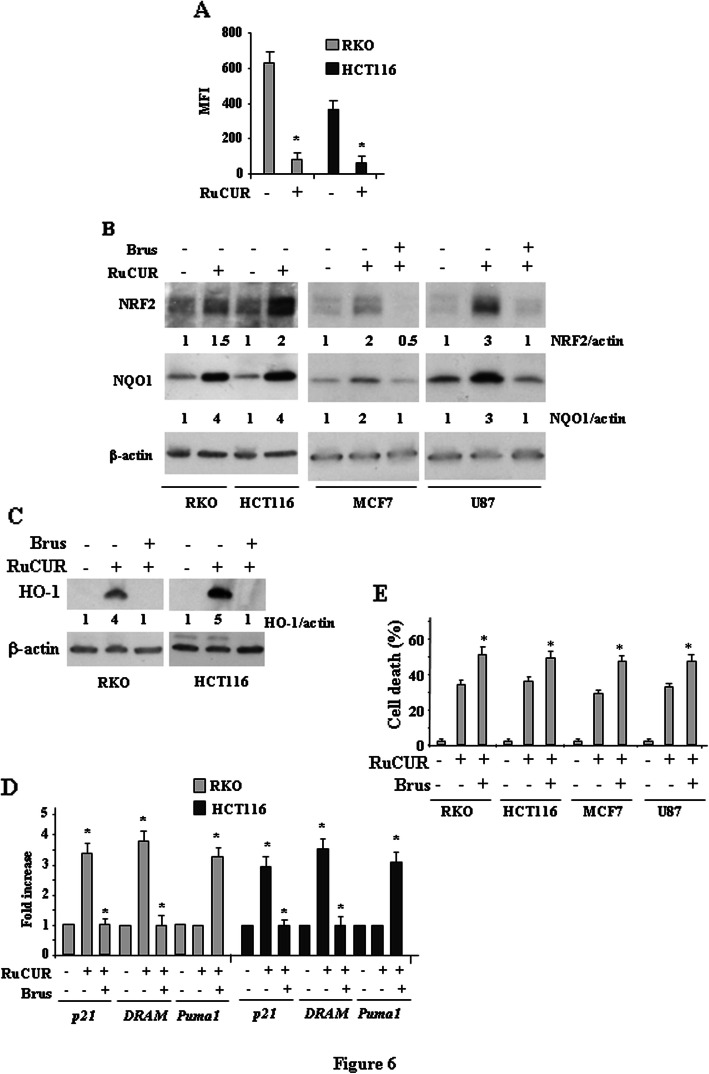
RuCUR compound induces NRF2 pathway in wtp53-carrying cancer cells. (**a**) Oxidant species production in RKO and HCT116 cells after RuCUR (100 μM) treatment for 16 h evaluated by 2′,7′-dichlorofluorescein diacetate (DC-FDA) staining and assessed by Fluorescence-Activated Cell Sorting (FACS) analysis. Histograms of the mean fluorescence intensity (MFI) represent the mean ± SD. * (*p* ≤ 0.01). (**b**) Western blot analysis of the indicated protein levels was performed in (left panel) RKO and HCT116 cells untreated or treated with RuCUR (100 μM) for 24 h and in MCF7 and U87 cells (right panel) cells pre-treated with brusatol (100 nM for 4 h) prior to adding RuCUR (100 μM) for 24 h. Actin was used as protein loading control. The ratio of the protein levels vs β-actin, following densitometric analysis using ImageJ software, is reported (**c**) Western blot analysis of the indicated protein levels in RKO and HCT116 cells pre-treated with brusatol (100 nM for 4 h) prior to adding RuCUR (100 μM) for 24 h. Actin was used as protein loading control. The ratio of protein levels vs β-actin, following densitometric analysis using ImageJ software, is reported. (**d**) Total mRNA was extracted from RKO and HCT116 cells pre-treated with brusatol (100 nM for 4 h) prior to adding RuCUR (100 μM) for 24 h. The indicated gene expression was assayed by the polymerase chain reaction (PCR) of reverse-transcribed cDNA. Densitometric analysis was performed using ImageJ software to calculate the gene/28S ratio. Histograms represent the fold increase quantified with respect to controls set to 1.0, ± SD. * (*p* ≤ 0.01). (**e**) Cell viability was measured by trypan blue exclusion assay in RKO and HCT116 cells pre-treated with brusatol (100 nM for 4 h) prior to adding RuCUR (100 μM) for 24 h and expressed as cell death percentage ± S.D. * (*p* ≤ 0.01) (single treatments compared to untreated cells)

Finally, we evaluated the effect of RuCURC on NRF2 pathway in wtp53 cells. We found that RuCUR reduced the intracellular ROS generation (Fig. 6a) that correlated with increased NRF2 pathway (Fig. 6b). Inhibition of NRF2 activity by brusatol strongly impaired the RuCUR-induced NQO1 and HO-1 levels (Fig. 6b and c). Intriguingly, brusatol co-treatment impaired the RuCUR-induced p21 and DRAM expression, while increased Puma expression (Fig. 6d), suggesting that inhibiting NRF2 could reestablish p53 apoptotic activity. In line with this effect, NRF2 inhibition further increased RuCUR-induced cell death (Fig. 6e).

**Fig. 7:**
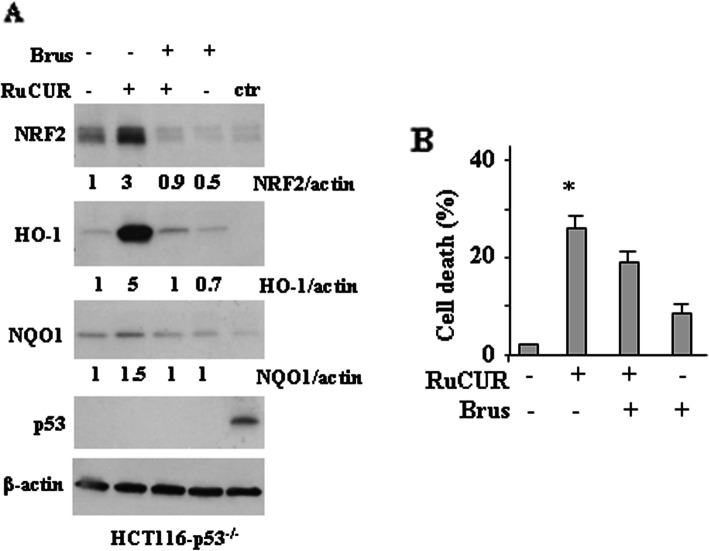
RuCUR compound treatment in p53 null cells. (**a**) Western blot analysis of the indicated protein levels was performed in HCT116 p53−/−cells pre-treated with brusatol (100 nM for 4 h) prior to adding RuCUR (100 μM) for 24 h. Actin was used as protein loading control. The ratio of the protein levels vs β-actin, following densitometric analysis using ImageJ software, is reported. Ctr: positive control for p53. (**b**) Cell viability was measured by trypan blue exclusion assay in HCT116 p53−/−cells pre-treated with brusatol (100 nM for 4 h) prior to adding RuCUR (100 μM) for 24 h and expressed as cell death percentage ± S.D. * (*p* ≤ 0.01) (single treatments compared to untreated cells)

As a final point, to evaluate the role of endogenous p53 we used HCT116 p53 null cells. We found that RuCUR activated NRF2 pathway also in p53 null cells (Fig. 7a); however, analysis of cell viability show that the RuCUR-induced cell death was slightly reduced by NRF2 inhibition with brusatol (Fig. 7b), opposite to what observed in mutp53 and wtp53 cells (Figs. 5 and 6a, e). Altogether, these findings suggest that reducing NRF2 pathway in wtp53-carrying cells may unbalance the pro-survival/pro-death axis toward cell death and restore p53 apoptotic activity that indeed is crucial for cancer cell death; on the other hand, reducing NRF2 pathway in p53 null cells did not significantly increase cancer cell death, underlining the critical role of p53 in cell death.

## Discussion

**Fig. 8:**
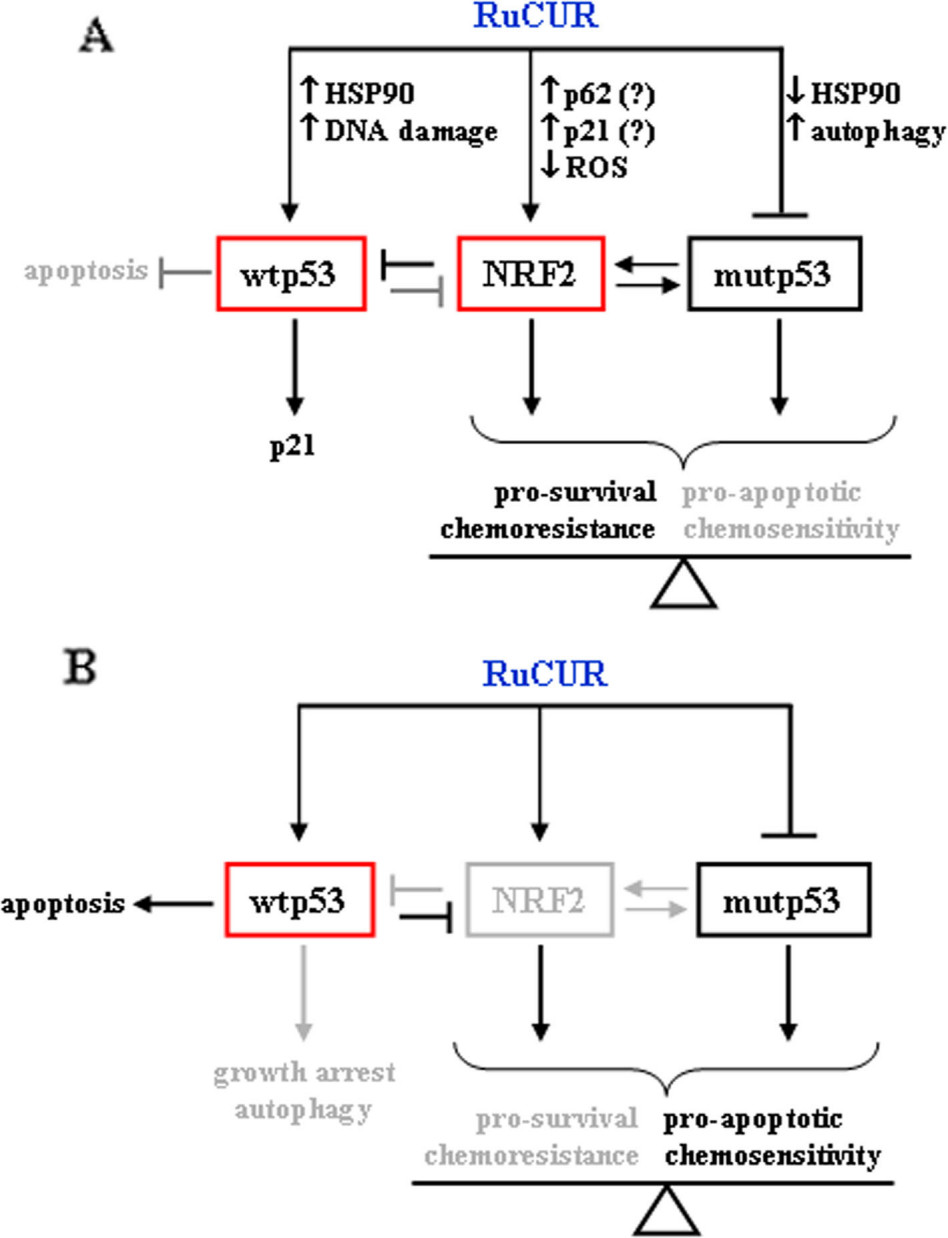
Schematic representation of the effect of RuCUR in cancer cells. (**a**) RuCUR induced NRF2, degraded mutp53 and induced wtp53, although not the apoptotic activity. (**b**) NRF2 inhibition restored wtp53 apoptotic activity and further degraded mutp53, unbalancing the cell-fate toward cell death

The findings of the present study revealed that a novel water-soluble ruthenium(II)-curcumin compound (RuCUR) [42] was able to induce cancer cell death that correlated with mutp53 downregulation and with activation of wtp53; they also revealed a resistance mechanism via the NRF2-induced antioxidant system likely enhanced by ROS reduction. In addition, our findings suggest that NRF2 inhibitors can overcome the RuCUR resistance via inhibition of the antioxidant system (Fig. 8a, b). However, the lack of p53 did not contribute to counteract the death resistance even following NRF2 inhibition, underlying the important role of p53 (re)activation for cancer cells demise.

Mutp53 proteins may promote tumor invasion, metastasis and chemoresistance, disclosing mutp53 as a guardian of the cancer cells [59]. Restoration of wild-type p53 function prompts the rapid elimination of human cancers carrying a functional loss of p53, often by gene mutation [7–9]. Many p53-reactivating compound have been shown to induce anti-tumor effects in several tumor types [60]. A number of evidences are now supporting the role of autophagy in mutp53 degradation. Autophagy is a catabolic process that helps to eliminate unfolded proteins or damaged organelles, promoting cell survival [61, 62] even if, in some instances, it may induce cell death [63]. Besides mutp53, other oncogenic proteins are degraded via autophagy including BCR-ABL, PML-RAR, Ret, KIT and Myc, consistent with the tumor-suppressive activity of autophagy [64, 65]. Therefore, inducing autophagy may contribute to cancer cell death and to disable oncogenes but it can also have possible disadvantages due to autophagy crosstalk with the immune response which is fundamental for the success of anticancer therapies [65, 66].. Interestingly, a mutual interplay between autophagy and mutp53 may occur: while autophagy degrades mutp53, mutp53 inhibits autophagy counteracting its own elimination as a self-protecting mechanism, thus promoting chemoresistance [67]. Curcumin has been shown to induce autophagy [38] and to contribute to mutp53 degradation [16–18, 37]. In line with this findings, here we found that RuCUR triggered mutp53 downregulation likely through autophagy; in addition, it also induced HSP90 reduction, a molecular chaperone critical for maintaining mutp53 stability [4] protecting mutp53 from MDM2-induced degradation [68]. Curcumin has been shown to have an indirect effect on HSP90 downregulation [54], although the exact mechanism of HSP90 reduction in our setting needs to be further explored. Interestingly, HSP90 chaperoning activity can target also wtp53, promoting its transcriptional activity [53], underscoring the two-faced role of molecular chaperones in p53 activity [69]. Thus, here we found that RuCUR increased HSP90 levels in wtp53 cells that correlated with p53 activation, although the mechanism of HSP90/wtp53 regulation has not been clearly elucidated yet.

Curcumin may induce NRF2 pathway activation [36]. NRF2 induces cytoprotective genes that, on one hand, protect cells from the oxidative stress [28] and, on the other hand, induce cancer cells proliferation, resistance to drugs, and apoptosis inhibition [70]. NRF2 may also interact with mutp53 [56, 58] suggesting a criminal alliance to sustain cancer cells [71]. Here, we found that RuCUR induced NRF2 stabilization in all cell lines, regardless of the p53 status. Several mechanisms can correlate with NRF2 stabilization such as ROS inhibition, increased p62/SQSTM1 [31] or p21^Cip1/WAF1^ levels [32]. We found that RuCUR reduced ROS generation in both wtp53 and mutp53 cells, it also induced p62 in mutp53 cells and p21 in wtp53 cells, although the exact mechanisms of NRF2 stabilization in our setting need to be further explored. Interestingly, NRF2 inhibition increased cell death in both wtp53 and mutp53 cells, underscoring the resistance effect of the antioxidant system. At molecular level, NRF2 inhibition further decreased mutp53 levels, underscoring the link between NRF2 and mutp53 to sustain cancer cell survival (Fig. 8). NRF2 inhibition in wtp53 cells impaired the RuCUR-induced p21 and DRAM expression, while increased Puma expression, suggesting that inhibiting NRF2 could reestablish p53 apoptotic activity [48, 52]. The mechanism of NRF2 inhibition of the p53 apoptotic function is an interesting filed of study and we hypothesize that NRF2 and the oxidative system may impair the function of the p53 apoptotic activator homeodomain-interacting protein kinase 2 (HIPK2) [72–75], although further studies are needed to demonstrate this hypothesis. It is worth to note that p53 apoptotic activation in turn inhibits NRF2 cytoprotective function that may hamper the p53-induced apoptosis [76], in a complex regulatory loop between NRF2 and p53 (Fig. 8). Finally, reduction of the antioxidant system increased cell death only in mutp53 and wtp53 cells while it did not have this effect in p53 null cells. These findings underscore the critical role of p53 for efficient cancer cell death, although the exact mechanisms need to be further explored.

## Conclusions

This study suggests that the overexpression of the antioxidant system is involved in the mechanism of cancer cell resistance to RuCUR treatment. The elimination of mutp53 and the activation of wtp53 induced cell death although only after inhibition of the antioxidant system cell death greatly improved. Interestingly, p53 null cells did not undergo increased cell death following inhibition of the NRF2 pathway, underscoring the important role of p53 in cancer cell death. These findings also suggest that the use of phitochemicals that can increase the antioxidant system and, for this reason, being useful antiaging agents, may not be beneficial for the demise of tumors, despite autophagy induction targeting oncogenes such as mutp53, underlining the complex relationship between autophagy, antioxidant system and tumor cell death. In conclusion, these findings may represent a paradigm for better understanding the complex interplay between NRF2 and p53 molecular pathways, in order to design more efficient anticancer therapies. Further preclinical and clinical investigations of RuCUR /NRF2 inhibition combination should be performed in order to explore the anticancer activities.

## Data Availability

All data generated or analysed during in this study are included in this published article.

## Abbreviations

3D: Three-dimensional
BSA: Bovine serum albumin
DCFDA: Dichlorofluorescein diacetate
DMEM: Dulbecco modified Eagle’s medium
FBS: Foetal bovine serum
GSH: Glutathione
HIF-1: Hypoxia-inducible factor 1
HIPK2: Homeodomain-interacting protein kinase 2
HO-1: Heme-oxygenase 1
HSP: Heat shock protein
KEAP1: Kelch-like ECH-associated protein 1
MFI: Mean fluorescence intensity
mTOR: Mammalian target of rapamycin
mutp53: Mutant p53
NQO1: NAD(P)H quinone oxidoreductase 1
NRF2: Nuclear factor erythroid 2-related factor 2
PARP: Poly(ADP-ribose) polymerase
PVDF: Polyvinylidene difluoride
ROS: Reactive oxygen species
RT-PCR: Reverse-Transcribed Polymerase Chain Reaction
SD: Standard deviation
SOD: Superoxide dismutase
wtp53: Wild-type p43
